# Interpretation of serial interferon-gamma test results to measure new tuberculosis infection among household contacts in Zambia and South Africa

**DOI:** 10.1186/s12879-020-05483-9

**Published:** 2020-10-15

**Authors:** Rosa Sloot, Kwame Shanaube, Mareli Claassens, Lily Telisinghe, Ab Schaap, Peter Godfrey-Faussett, Helen Ayles, Sian Floyd

**Affiliations:** 1 Desmond Tutu TB Centre, Department of Paediatrics and Child Health, Faculty of Medicine and Health Sciences, Stellenbosch University, Cape Town, South Africa; 2 Zambart, School of Medicine, University of Zambia, Lusaka, Zambia; 3 School of Social and Community Medicine, University of Bristol, Bristol, UK; 4 UNAIDS, Geneva, Switzerland; 5 Clinical Research Department, London School of Hygiene and Tropical Medicine, London, UK; 6 Department of Infectious Disease Epidemiology, London School of Hygiene and Tropical Medicine, London, UK

**Keywords:** Conversion, Tuberculosis, QuantiFERON, Interferon-gamma

## Abstract

**Background:**

A more stringent QuantiFERON-TB Gold In-Tube (QFT) conversion (from negative to positive) definition has been proposed to allow more definite detection of recent tuberculosis (TB) infection. We explored alternative conversion definitions to assist the interpretation of serial QFT results and estimate incidence of TB infection in a large cohort study.

**Methods:**

We used QFT serial results from TB household contacts aged ≥15 years, collected at baseline and during two follow-up visits (2006–2011) as part of a cohort study in 24 communities in Zambia and South Africa (SA). Conversion rates using the manufacturers’ definition (interferon-gamma (IFN-g) < 0.35 to ≥0.35, ‘def1’) were compared with stricter definitions (IFN-g < 0.2 to ≥0.7 IU/ml, ‘def2’; IFN-g < 0.2 to ≥1.05 IU/ml, ‘def3’; IFN-g < 0.2 to ≥1.4 IU/ml, ‘def4’). Poisson regression was used for analysis.

**Results:**

One thousand three hundred sixty-five individuals in Zambia and 822 in SA had QFT results available. Among HIV-negative individuals, the QFT conversion rate was 27.4 per 100 person-years (CI:22.9–32.6) using def1, 19.0 using def2 (CI:15.2–23.7), 14.7 using def3 (CI:11.5–18.8), and 12.0 using def4 (CI:9.2–15.7). Relative differences across def1-def4 were similar in Zambia and SA. Using def1, conversion was less likely if HIV positive not on antiretroviral treatment compared to HIV negative (aRR = 0.7, 95%CI = 0.4–0.9), in analysis including both countries. The same direction of associations were found using def 2–4.

**Conclusion:**

High conversion rates were found even with the strictest definition, indicating high incidence of TB infection among household contacts of TB patients in these communities. The trade-off between sensitivity and specificity using different thresholds of QFT conversion remains unknown due to the absence of a reference standard. However, we identified boundaries within which an appropriate definition might fall, and our strictest definition plausibly has high specificity.

## Background

The impact of tuberculosis (TB) preventive treatment is largely determined by adequate identification and diagnosis of individuals with an increased risk of progression from latent TB infection (LTBI) to clinical disease. Both the tuberculin skin test (TST) and two commercial interferon gamma (IFN-g) release assays (IGRA), QuantiFERON-TB Gold In-Tube (QFT) and T-SPOT.TB assay, do not have a high accuracy for the prediction of active TB and cannot differentiate between a previously acquired and new TB infection [1, 2]. However, the IGRA offers a potential method of serial testing to detect new TB infection and target high-risk individuals for preventive treatment. Unlike the TST it can be repeated without sensitization and boosting in subsequent tests, and it has better specificity than that of TST in one-time screening [3, 4]. Several studies have indeed shown that TB progression risk is higher among those that recently converted, i.e., from a negative to a positive IGRA result, compared to those who remained IGRA negative on repeated testing [5, 6].

Since the Centers for Disease Control and Prevention (CDC) has recommended QuantiFERON-TB Gold (QFT) for baseline and serial testing [4], there is growing evidence that IFN-g levels bordering the manufacturer’s recommended assay cutoff of 0.35 IU/ml are more likely to show discordant results upon serial testing [7–12]. These patterns are seen in settings with varying TB burden, suggesting that at least some of the sources of IGRA variability are immunological (i.e., boosting and modulation) or due to assay reproducibility issues, independent of the risk of exposure [11, 12]. Considering the dynamic characteristics of IFN-g responses over time, a simple dichotomous definition might not be appropriate. Hence, several studies have suggested the introduction of a ‘zone of uncertainty’ [5, 13–16] to assist in distinguishing new TB infections from non-specific variation. It has been proposed that this zone should lie between 0.2 and 0.7 IU/ml to allow a more definitive detection of recent TB infection and reduce the risk of unnecessary initiation of preventive treatment in settings where IGRA is used [17–20]. To date, only one large longitudinal study, conducted by Nemes et al., has provided sound evidence which supports the use of a stringent QFT conversion definition using the proposed zone of uncertainty [17]. More evidence is needed from larger cohort studies and in other settings to inform guidelines on serial QFT testing.

In this study we explored the use of the proposed zone of uncertainty (between 0.2 and 0.7 IU/ml) and several alternatives, to provide “boundaries” to assist in the interpretation of serial QFT results and estimate incidence of TB infection in a large cohort study. We use epidemiological and clinical data collected among household TB contacts during multiple years of follow-up as part of a large community randomized trial, the Zambia South Africa TB and AIDS Reduction (ZAMSTAR) trial, carried out from 2005 to 2011 in 24 communities (16 in Zambia, 8 in the Western Cape, South Africa) [21].

## Methods

### Study setting

The primary aim of the ZAMSTAR trial was to measure the effect of household and community interventions on TB prevalence and the incidence of new infection with *M. tuberculosis* in the general population, described elsewhere [21, 22]. Secondary outcomes in ZAMSTAR were TB transmission within the households of TB patients, and cumulative incidence of TB disease in household contacts of TB patients [23]. We conducted a retrospective analysis using secondary outcome data collected among the household members of newly diagnosed TB cases. Households were recruited in each of the 24 ZAMSTAR communities. Study communities, urban, peri-urban and rural, were selected based on TB notification rates greater than 400/100,000 per annum and having an HIV seroprevalence higher than estimated for the whole country (Zambia) or province (Western Cape) [21]. Findings from a 2010 HIV prevalence survey in study communities estimated a seroprevalence of approximately 15% in Zambia and 20% in South Africa [22].

### Study population

Secondary outcomes at household level were measured among a cohort of adult TB patients and their household members. After the start of the ZAMSTAR trial, TB patients, subsequently referred to as index patients, were recruited within 1 month of initiating TB treatment at government TB diagnostic health facilities. The index patient was asked for permission to visit his/her household, and if they gave permission then the household was visited shortly afterwards and household members (aged < 5 years and ≥ 15 years) were invited to participate. Household members were defined as individuals who usually slept in the home, ate with the index patient and who identified a common household head [23].

### Data collection

**Fig. 1:**
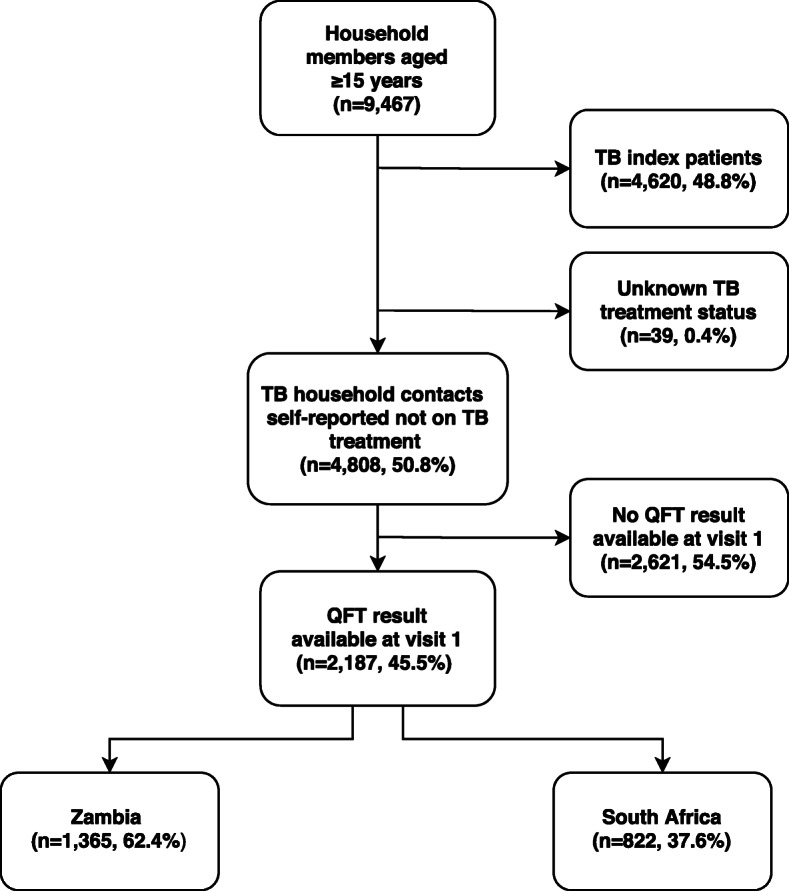
Flow chart household contacts eligible for analysis

The retrospective analysis in this study used epidemiological and clinical data collected among the household members who consented to participate, aged ≥15 years and reported not to be on TB treatment at baseline (visit 1), subsequently referred to as household contacts. Household contacts were visited from September 2006 to January 2011 and QFT tests were conducted from January 2007. Hence, a large proportion of recruited contacts did not have a QFT result available at visit 1 (Fig. 1). Data were collected at visit 1 (January 2007–August 2008), and during two follow-up measurements: visit 2 (September 2008–February 2010) and visit 3 (July 2010–January 2011). Median follow-up time was 16 months (range 11–21) between visit 1-visit 2, and 18 months (range 15–20) between visit 2-visit 3.

Household contacts were asked to respond to a structured questionnaire during household visit 1. Data on sex, age, marital status, education level, employment status, smoking history, alcohol use, drug use, and experience of TB and HIV treatment were collected. Data from TB index patients (sex, age and HIV status) were linked to contact data through a common household number if written informed consent was obtained. Additional clinical index characteristics were obtained if the index patient could be linked to the national TB register, and included type of TB (pulmonary, extrapulmonary, and smear status), and index patient type (transferred, relapsed, new, resumed). Remaining data collected for each household included: household wealth status (based on an asset index), baseline TST prevalence region, and whether the household was part of the ZAMSTAR household intervention. The covariate baseline TST prevalence region, a marker of TB infection prevalence in the general community, was established from TST prevalence surveys conducted among primary school children in all 24 communities at the start of the ZAMSTAR trial [22, 24]. These surveys were used to characterize ZAMSTAR communities, regarding baseline TST prevalence. The covariate household intervention (yes or no) represents the outcome of the randomisation of ZAMSTAR communities into intervention arms. Two trial interventions were delivered between 2006 and 2009 and included community-based enhanced case finding for tuberculosis (ECF intervention) and household counselling and provision of combined TB/HIV prevention services at the household level (HH intervention). The interventions were randomised in a factorial design so that 6 communities received standard-of-care, 6 ECF alone, 6 HH counselling alone, and 6 both ECF and HH interventions [21].

During each visit (visit 1, visit 2, visit 3), a venous blood sample was collected for laboratory HIV testing and QFT testing among household contacts who consented to participate and also to provide a blood sample. TSTs were also performed, independent of positivity during previous visits. HIV testing was done using the Abbot Murex HIV Ag/Ab combination ELISA (Murex Biotech, Dartford, United Kingdom). TB infection was measured by TST and QFT at baseline and during follow-up. The TST was conducted using 2 TU (Tuberculin Units) of purified protein derivative RT23 with Tween, supplied by the Statens Serum Institut (Copenhagen, Denmark). A dose of 0.1 ml was injected intradermally on the left forearm. Skin reactions were read using calipers 72 h later. TST and QFT tests were performed according to the manufacturer’s instructions. Blood for QFT was drawn before TST was administered usually on the same day.

### Study outcomes

Our main outcomes were QFT positivity at visit 1 and QFT conversion at visit 2/3. Published literature and the distribution of IFN-g values in our study were investigated to define definitions of QFT positivity and QFT conversion with different IFN-g assay cutoffs. QFT results in our data were expressed as IFN-g concentration IU/ml. This was calculated as TB antigen (TBAg) response minus the assays’ negative control response (Nil). QFT cannot accurately measure absolute IFN-g values greater than 10 IU/ml, therefore such values were treated as 10 IU/ml. Indeterminate outcomes were excluded from the analyses.

#### Baseline QFT positivity

Previous studies questioned the use of the manufacturers cut-off for a positive QFT result (IFN-g ≥ 0.35) and have proposed using a ‘zone of uncertainty’ between 0.2 and 0.7 IU/ml. We explored the use of this zone by plotting histograms to visualize the distribution of IFN-g IU/ml at visit 1. Histograms were plotted among HIV negative household contacts as immunosuppression by HIV infection can result in a diminished antigen response, resulting in a low negative predictive value of the IGRA in HIV positive individuals [25].

#### QFT conversion

Incidence rates of QFT conversion (from a negative QFT result at visit 1 to a positive QFT result at either visit 2 or visit 3) were calculated using the QFT conversion definition as suggested by previous studies (IFN-g < 0.2, ≥0.7 IU/ml). These rates were compared with incidence rates using stricter QFT conversion definitions. Histograms were plotted of the absolute IFN-g distribution at visit 2 among contacts with a negative QFT at visit 1, and of the distribution of change in IFN-g between visit 1 and visit 2 to help to determine stricter conversion definitions. All histograms were plotted among HIV negative household contacts, unless stated otherwise.

### Statistical analysis

#### Baseline QFT positivity

TB household contacts were eligible for baseline analysis if they had a visit 1 QFT result available and if they self-reported not on TB treatment. The prevalence of infection was defined as the number of QFT positive results among the total number of individuals with a positive or negative result. The strength of the relationship between individual and household characteristics and QFT positivity was assessed with random effects logistic regression. The random effects approach specified the household of residence as the clustering variable in univariable and multivariable analysis. Two different multivariable models were developed. The first multivariable model included a priori selected contact- and household characteristics: sex, age, HIV status, household intervention (Y/N) and TST prevalence region. In this model all factors were added simultaneously. The second multivariable model was built using forward selection and assessed the relationship between all available contact-, index-, and household characteristics with the outcome, guided by the strength of evidence for the association with the outcome. First, factors significantly associated with QFT positivity (overall *p*-value< 0.05) in univariable analysis were simultaneously added to the a priori model (including sex, age, HIV status, and TST prevalence region). Second, only factors that showed evidence of association after adjustment for a priori factors were included in the subsequent model building step. In this step factors were added one by one to assess the association between each characteristic and outcome using the Wald test. Only factors that showed evidence of association were included in the final multivariable model. Unadjusted and adjusted odds ratios (ORs) and 95% confidence intervals (CIs) were presented for each risk factor. Analysis was performed for both countries combined, and separately for Zambia and South Africa.

#### Conversion analysis

Contacts were eligible for QFT conversion analysis if their QFT result was negative at visit 1 and if they had a positive or negative result available at visit 2 and/or visit 3. Contacts were excluded from analysis if they had a missing QFT result at both visit 2 and visit 3. Incidence rates (IRs) and 95% CIs were reported as number of conversion events per 100 person-years of follow-up time. The date of visit 1 was used as the start of follow-up of the household contact. Follow-up time ended halfway between visit 1 and 2 if conversion occurred at visit 2, or halfway between visit 2 and visit 3 if QFT was negative at visit 2 and conversion occurred at visit 3. End follow-up time of contacts who did not convert was placed at the date of the last available negative QFT test result. Visit 2 dates were imputed for those with missing QFT values at visit 2 and a valid value at visit 3. Dates were imputed as the median date of available visit 2 dates, stratified by community (24 communities). Analysis time was split into two time-bands (visit 1- visit 2 and visit 2- visit 3) to compare QFT conversion rates between visit 1–2 and visit 2–3. Additionally, IRs of the analysis that included all contact outcomes were compared to the IRs of the analysis restricted to contacts who had QFT known at visit 2.

The strength of the relationship between individual and household characteristics and QFT conversion was assessed using Poisson regression analysis in a similar way as described for baseline logistic regression analysis (with an a priori model, and forward selection). Unadjusted and adjusted rate ratios (RRs) and 95%CIs were presented for each risk factor in the a priori model. Risk factors in the forward selection model were only presented if associated with the outcome. Analyses were done for both countries combined, and separately for Zambia and South Africa. All analyses were completed in Stata (version 14.0; Stata Corp, College Station, TX).

## Results

### Study population

Nine thousand four hundred sixty-seven household members aged ≥15 years gave consent to participate at visit 1 in the year 2007 (Fig. 1). Among these were 4808 (51%) household contacts not on TB treatment. Contacts with a QFT result available at visit 1 were eligible for analysis (2187/4808, 46%). These contacts did not differ (sex, age, HIV status) from the 2621 contacts that did not have a QFT result available at visit 1 (Fig. 1). Among household contacts eligible for analysis, 1365 (62%) contacts were recruited from 805 households in Zambia, and 822 (38%) contacts were recruited from 474 households in South Africa.

Of the 1365 household contacts in Zambia, 1164 had a valid (positive or negative) QFT test result and 201 were indeterminate (using manufacturers’ definition, Table 1). In South Africa, 804 contacts had a valid (positive or negative) QFT test result and 18 were indeterminate (using manufacturers’ definition, Table 1). In both Zambia and South Africa the majority were female, aged between 15 and 24 years and were HIV negative (Table A1, additional file). One thousand two hundred twenty-two of 1968 contacts had TB status available at one or both follow-up visits. In total, 57 (5%) of the 1222 contacts developed TB during follow-up, with only a minority (17/57, 30%) diagnosed after visit 2, so we did not use TB incidence outcomes to inform conversion definitions. Median time between TB diagnosis and the most recently available prior QFT test result was 9 months (IQR = 4–15). Fig. A1 (additional file) shows the distribution of these IFN-g results (prior to TB diagnosis) among 57 household contacts who developed TB during follow-up, and Fig. A2 (additional file) shows the IFN-g distribution among 1165 contacts who did not develop TB. Contacts who developed TB had a higher median IFN-g response prior to diagnosis, were older, and a higher proportion were HIV-positive, compared with the contacts who did not develop TB (Table A2, additional file).

**Table 1:** Overview household contacts eligible for baseline analysis using different definitions of visit 1 QFT status

		Definition 1 (≥0.2)			Definition 2^**a**^ (≥0.35)			Definition 3 (≥0.7)	
	Definition 1 (≥0.2 IU/ml)	Zambia n (%)	South Africa n (%)	Definition 2 (≥0.35 IU/ml)^**a**^	Zambia n (%)	South Africa n (%)	Definition 3 (≥0.7 IU/ml)	Zambia n (%)	South Africa n (%)
**Positive**		735 (54)	626 (76)		669 (49)	574 (70)		599 (44)	504 (61)
Nil	≤8.0			≤8.0			≤8.0		
TBAgNil	≥0.2 & (≥25% of Nil value)			≥0.35 & (≥25% of Nil value)			≥0.7 & (≥25% of Nil value)		
**Negative**		445 (33)	182 (22)		495 (36)	230 (28)		551 (40)	297 (36)
Nil	≤8.0			≤8.0			≤8.0		
TBAgNil	< 0.2 or < 25% Nil			< 0.35 or < 25% Nil			< 0.7 or < 25% Nil		
MitogenNil	≥0.5			≥0.5			≥0.5		
**Indeterminate**^**b**^	all other values	185 (14)	14 (2)	all other values	201 (15)	18 (2)	all other values	215 (16)	21 (3)

Footnote: *Nil* negative control response; *TBAgNil* antigen response minus negative control response; *MitogenNil* positive control response minus negative control response

^a^ Manufacturer’s recommended assay cut-off

^b^ The number of indeterminate test results differs depending on the QFT definition used: if a positive value becomes a negative value using a stricter QFT definition, it will only be quantified as negative if the value is accompanied with MitogenNil≥0.5. If not, the value is quantified as indeterminate

### Determining QFT test cut-offs

**Fig. 2:**
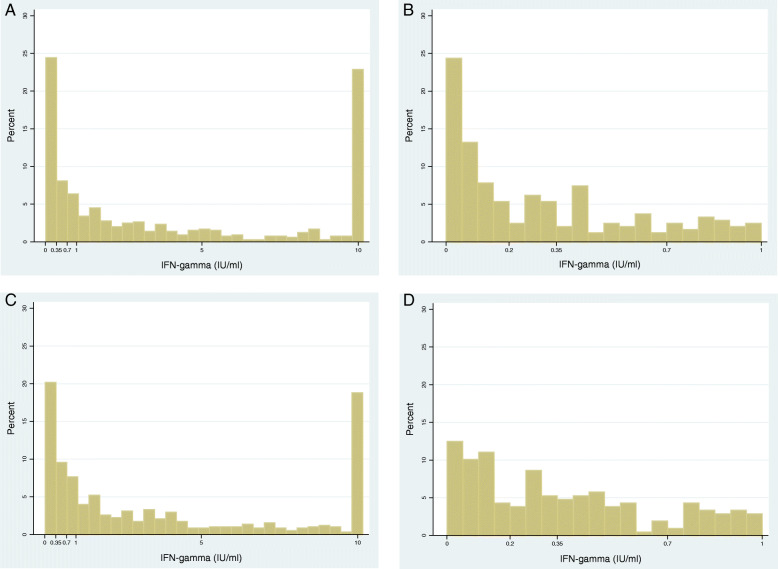
Distribution visit 1 IFN-gamma results among HIV negative household contacts with a valid IFN-gamma value. **a**. Zambia, all results (*n* = 797); **b**. Zambia, zoomed-in (0–1 IU/ml); **c**. South Africa, all results (*n* = 605); **d**. South Africa, zoomed-in (0–1 IU/ml). Baseline definition 2 (Table 1) was used to identify contacts with a valid (positive or negative) IFN-g value at visit 1

Quantitative IFN-g values of the QFT results at visit 1 were plotted in histograms (Fig. 2) and showed the absence of any natural quantitative breakpoint in both countries (Fig. 2b and d). Therefore, stricter definitions to the manufacturer’s definition of QFT positivity (IFN-g ≥ 0.35 IU/ml) were based on a conventional approach; we chose multiples of 2, 3, and 4 times the manufacturer’s definition and used 0.7, 1.05, and 1.4 as cut-offs to define positive versus negative responses.

**Fig. 3:**
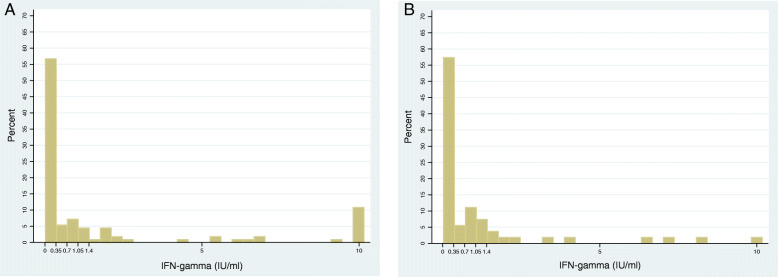
Distribution IFN-gamma results at visit 2 among HIV negative household contacts with a negative QFT result at visit 1. **a**. Zambia, all results (*n* = 172); **b**. South Africa, all results (*n* = 63). Baseline definition 2 (Table 1) was used to select contacts with a negative QFT result (< 0.35 IU/ml) at visit 1. No rules were applied to visit 2; all valid (positive and negative) IFN-g values were included

TB infection prevalence in our cohort was described using a stricter definition (IFN-g ≥ 0.7 IU/ml) and a less strict definition (IFN-g ≥ 0.2 IU/ml) of baseline QFT positivity, to provide “boundaries” to our estimates (Table 1). Plotting the quantitative values of the QFT results at visit 2 among contacts with a negative QFT result (< 0.35 IU/ml) at visit 1 (Fig. 3) show that the majority of contacts negative at visit 1 remained negative (< 0.35) at visit 2, in both Zambia (124/172, 72%) and South Africa (40/63, 63%). The remaining IFN-g values did not show a clear “breakpoint” between 0.35–1.4 IU/ml (Fig. 3).

Based on literature, and findings in Fig. 3, we explored the following alternatives to the manufacturer’s conversion definition (< 0.35, ≥0.35 IU/ml, definition 1): a negative result at visit 1 was defined as < 0.2 IU/ml and conversion during follow-up as either ≥0.7 (definition 2), ≥1.05 (definition 3), or ≥ 1.4 (definition 4) IU/ml (Table 2).

**Table 2:** Definitions used to determine QFT conversion at visit 2 and visit 3

	Conversion definition 1^**a**^ (< 0.35, ≥0.35)	Conversion definition 2 (< 0.2, ≥0.7)	Conversion definition 3 (< 0.2, ≥1.05)	Conversion definition 4 (< 0.2, ≥1.4)
**Eligible for analysis if negative at visit 1**				
Nil	≤8.0	≤8.0	≤8.0	≤8.0
TBAgNil	< 0.35 **or** < 25% Nil	< 0.2 **or** < 25% Nil	< 0.2 **or** < 25% Nil	< 0.2 **or** < 25% Nil
MitogenNil	≥0.5	≥0.5	≥0.5	≥0.5
**Positive at visit 2 and/or visit 3**				
Nil	≤8.0	≤8.0	≤8.0	≤8.0
TBAgNil	≥0.35 **&** ≥ 25% of Nil **&** absolute increase of ≥0.35 IU/mlover baseline value	≥0.7 **&** ≥ 25% of Nil	≥1.05 **&** ≥ 25% of Nil	≥1.4 **&** ≥ 25% of Nil
**Negative at visit 2 and/or visit 3**				
Nil	≤8.0	≤8.0	≤8.0	≤8.0
TBAgNil	< 0.35 **or** < 25% Nil	< 0.7 **or** < 25% Nil	< 1.05 **or** < 25% Nil	< 1.4 **or** < 25% Nil
MitogenNil	≥0.5	≥0.5	≥0.5	≥0.5
**or**				
Nil	≤8.0			
TBAgNil	≥0.35 **&** ≥ 25% of Nil **&** absolute increase of < 0.35 IU/ml over baseline value			
MitogenNil	≥0.5			
**Indeterminate**	all other values	all other values	all other values	all other values

^a^Manufacturer’s recommended assay cut-off

Footnote: *Nil* negative control response, *TBAgNil* antigen response minus negative control response, *MitogenNil* positive control response minus negative control response

**Fig. 4:**
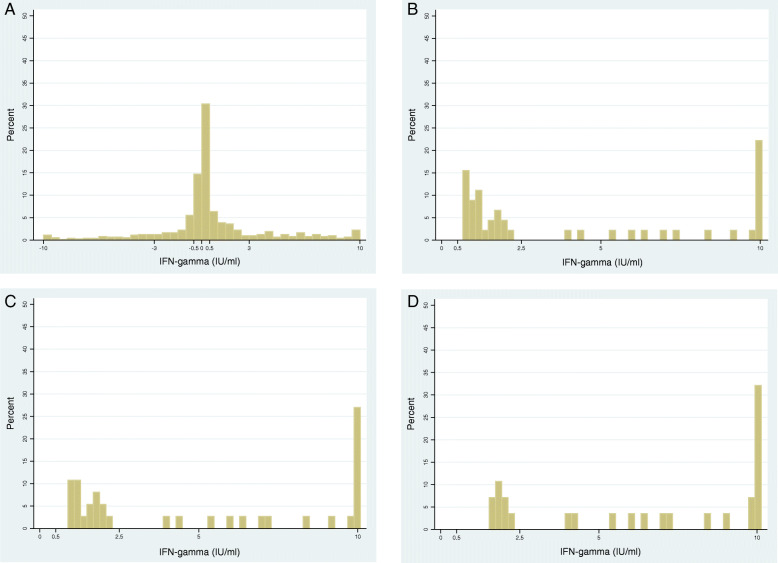
Distribution of change in IFN-gamma between visit 1 and visit 2 among HIV negative household contacts. **a-d** is shown for contacts (from both Zambia and South Africa) if they: **a.** had a valid QFT result available at visit 1 and visit 2; **b.** converted (< 0.2, ≥0.7); **c.** converted (< 0.2, ≥1.05); **d.** converted (< 0.2, ≥1.4). Baseline definition 2 (Table 1) was used to identify contacts with a valid (positive or negative) IFN-gamma value at visit 1 and 2

Figure 4 shows the distribution of change in IFN-g between visit 1 and visit 2 among HIV negative household contacts. 120/721 contacts (17%), who had a valid test result available at visit 1 and visit 2, had the same IFN-g value at both timepoints, 265 (37%) had a decrease in IFN-g, 99 (14%) had an IFN-g increase between 0 and 0.5 IU/ml, and 237 (33%) had an increase of IFN-g ≥ 0.5 IU/ml. Figure 4b shows that among 45 contacts who converted according to conversion definition 2 (IFN-g visit 1 < 0.2, visit 2 ≥ 0.7 IU/ml), 25 (56%) contacts had an IFN-g increase between 0.5–2.5 IU/ml, and the remaining 20 contacts had an increase ≥2.5 (44%). Figure 4c shows the distribution for 37 contacts who converted using definition 3 (IFN-g visit 1 < 0.2, visit 2 ≥ 1.05 IU/ml). 17 (46%) contacts had an IFN-g increase between 0.5–2.5 IU/ml, and the remaining 20 (54%) contacts had an increase ≥2.5. Corresponding figures among 28 contacts who converted using definition 4 (IFN-g visit 1 < 0.2, visit 2 ≥ 1.4 IU/ml) show: 8 (29%) had an increase between 0.5–2.5 IU/ml, and 20 (71%) an increase ≥2.5 (Fig. 4d).

### Baseline results

The proportion of household contacts who tested QFT positive at visit 1 using the three different definitions was higher in South Africa than in Zambia among HIV negative contacts (67–82% and 55–65%, respectively) (Table 3). In the analysis including both countries, the association between HIV status and QFT positivity was similar across definition 1–3. Contacts were less likely to be QFT positive if HIV positive; either if not on ARV (aOR:0.5, 95%CI:0.4–0.6) using definition 1–3, or if on ARV (aOR:0.4, 95%CI:0.2–0.8) using definition 1 and 3, and (aOR:0.4, 95%CI:0.3–0.8) using definition 2. The same direction of association for HIV status was found in both Zambia and South Africa, although evidence of association was weaker among HIV-positive individuals on ARV in South Africa, but numbers were small (Table 3). The proportion of household contacts who tested QFT positive at visit 1 was higher in communities with higher TST prevalence, visible across definitions and in both countries. Estimates of all variables in unadjusted analysis using definition 2 (Table A1, additional file) did not differ much from estimates in adjusted analysis (Table 3).

**Table 3:** Factors associated with a positive QFT result at visit 1 using different definitions of QFT positivity

	Definition 1			Definition 2^**a**^			Definition 3		
	(≥0.2 IU/ml)			(≥0.35 IU/ml)			(≥0.7 IU/ml)		
	QFT positive (n)	total (n)	Adjusted OR (95%CI)^**b**^	QFT positive (n)	total (n)	Adjusted OR (95%CI)^**b**^	QFT positive (n)	total (n)	Adjusted OR (95%CI)^**b**^
***Both countries***									
**Total**	1361 (68)	1988		1243 (63)	1968		1103 (57)	1951	
**HIV status**									
HIV negative	1026 (72)	1417	1	942 (67)	1402	1	839 (60)	1390	1
HIV positive, no ARV	288 (58)	496	0.5 (0.4–0.6)	258 (53)	491	0.5 (0.4–0.6)	229 (47)	489	0.5 (0.4–0.6)
HIV positive & ARV	30 (55)	55	0.4 (0.2–0.8)	28 (51)	55	0.4 (0.3–0.8)	21 (40)	53	0.4 (0.2–0.8)
Unknown	17 (85)	20		15 (75)	20		14 (74)	19	
***Zambia***									
**Total**	735 (62)	1180		669 (57)	1164		599 (52)	1150	
**HIV status**									
HIV negative	529 (65)	808	1	484 (61)	797	1	435 (55)	788	1
HIV positive, no ARV	176 (55)	321	0.5 (0.4–0.7)	158 (50)	316	0.5 (0.4–0.7)	142 (45)	314	0.5 (0.4–0.7)
HIV positive & ARV	18 (49)	37	0.4 (0.2–0.8)	16 (43)	37	0.4 (0.2–0.8)	12 (34)	35	0.3 (0.2–0.7)
Unknown	12 (86)	14		11 (79)	14		10 (77)	13	
**Region by TST prevalence**									
Lusaka, high TST	249 (68)	369	1	234 (64)	363	1	217 (60)	359	1
Urban, high TST	207 (63)	331	0.6 (0.4–0.9)	186 (57)	326	0.6 (0.4–0.9)	158 (49)	322	0.6 (0.4–0.9)
Urban, low TST	205 (57)	357	0.5 (0.4–0.8)	181 (52)	350	0.5 (0.4–0.7)	164 (48)	345	0.5 (0.4–0.7)
Rural, low TST	74 (58)	127	0.5 (0.3–0.9)	68 (54)	125	0.6 (0.3–0.9)	60 (48)	124	0.6 (0.3–0.9)
***South Africa***									
**Total**	626 (77)	808		574 (71)	804		504 (63)	801	
**HIV status**									
HIV negative	497 (82)	609	1	458 (76)	605	1	404 (67)	602	1
HIV positive, no ARV	112 (64)	175	0.4 (0.3–0.6)	100 (57)	175	0.4 (0.3–0.6)	87 (50)	175	0.4 (0.3–0.6)
HIV positive & ARV	12 (67)	18	0.7 (0.2–2.0)	12 (67)	18	0.7 (0.2–2.0)	9 (50)	18	0.7 (0.2–2.1)
Unknown	5 (83)	6		4 (67)	6		4 (67)	6	
**TST prevalence**									
High	366 (80)	460	1	338 (74)	457	1	298 (65)	455	1
Low	260 (75)	348	0.7 (0.5–1.0)	236 (68)	347	0.7 (0.5–1.0)	206 (60)	346	0.7 (0.5–1.0)

^a^Table A1, additional file, presents unadjusted odds ratios, estimates for sex and age, and *p*-values

^b^ Adjusted models also included sex, age and HH intervention (yes/no). All variables were simultaneously added to the regression models

Table A3 (additional file) presents index factors associated with QFT positivity (using definition 2). Household contacts were less likely QFT positive at visit 1 if the index patient had extra pulmonary TB (EPTB) compared to contacts with a pulmonary TB (PTB) smear positive index (aOR:0.6, 95%CI:0.4–0.9 including both countries, and aOR:0.5, 95%CI:0.3–0.8 for Zambia only). In Zambia, contacts were also less likely QFT positive if the index patient was PTB smear negative compared to having a PTB smear positive index (aOR:0.7, 95%CI:0.5–0.9) (Table A3). No association between index patient smear status or other index characteristics and QFT positivity was found in South Africa.

### Conversion results

Table A4 (additional file) shows the conversion analysis for the study population and how conversion status and follow-up time were calculated among household contacts with a negative QFT result at visit 1 using conversion definition 2. Identical principles were applied for the other three conversion definitions.

Conversion rates were similar between visit 1 and 2 compared to visit 2 and 3 in the analysis including both countries and in Zambia, and were lower between visit 2 and 3 compared to visit 1 and 2 in South Africa (Table 4). Among HIV-negative individuals, in the analysis including both countries, the QFT conversion rate was 27.4 per 100 person-years (95%CI:22.9–32.6) using definition 1, 19.0 using definition 2 (95%CI:15.2–23.7), 14.7 using definition 3 (95%CI:11.5–18.8), and 12.0 using definition 4 (95%CI:9.2–15.7). IRs were higher in Zambia than in South Africa. IRs were lower among HIV positive contacts not on ARV in the analysis including both countries: 15.8 (95%CI:11.7–21.4) definition 1, 12.3 (95%CI:8.6–17.6) definition 2, 9.7 (95%CI:6.5–14.4) definition 3, and 8.8 (95%CI:5.8–13.3) definition 4. Table 4 further shows that, irrespective of which conversion definition was used, similar HIV status patterns were observed in Zambia: HIV negative contacts had higher conversion rates compared to HIV positive contacts not on ARVs. In South Africa definition 2–4 showed different patterns (HIV positives not on ARV had higher IRs than HIV negatives), however numbers are small, and CIs wide. Conversion rates were higher in communities with higher levels of TST prevalence, visible in both countries. A sensitivity analysis was done restricted to contacts who had visit 2 QFT result known (Table A5, additional file) and showed comparable incidence rates to Table 4.

**Table 4:** Incidence rate QFT conversion at visit 2 and visit 3 using different definitions of conversion

	Definition 1 (< 0.35, increase 0.35) *n* = 439 negative at V1			Definition 2 (< 0.2, ≥0.7) *n* = 374 negative at V1			Definition 3 (< 0.2, ≥1.05) n = 374 negative at V1			Definition 4 (< 0.2, ≥1.4) n = 374 negative at V1		
	Incident events	Person years	Incident Rate (95%CI)	Incident events	Person years	Incident Rate (95%CI)	Incident events	Person years	Incident Rate (95%CI)	Incident events	Person years	Incident Rate (95%CI)
***Both countries***												
Total	175	7.5	23.2 (20.0–26.9)	114	6.8	16.7 (13.9–20.0)	93	7.0	13.1 (10.7–16.1)	79	7.2	10.9 (8.7–13.6)
*End follow-up V1-V2*	113	4.9	23.3 (19.3–27.9)	72	4.2	17.0 (13.5–21.5)	56	4.3	12.9 (9.9–16.8)	46	4.4	10.4 (7.8–13.9)
*End follow-up V2-V3*	62	2.7	23.1 (18.0–29.6)	42	2.6	16.1 (11.9–21.8)	37	2.7	13.5 (9.8–18.6)	33	2.8	11.6 (8.2–16.3)
HIV status contact*												
HIV negative	125	4.6	27.4 (22.9–32.6)	78	4.1	19.0 (15.2–23.7)	63	4.3	14.7 (11.5–18.8)	53	4.4	12.0 (9.2–15.7)
HIV positive, no ARV	42	2.7	15.8 (11.7–21.4)	30	2.4	12.3 (8.6–17.6)	24	2.5	9.7 (6.5–14.4)	22	2.5	8.8 (5.8–13.3)
HIV positive & ARV	6	0.3	20.2 (9.1–44.9)	5	0.3	18.1 (7.5–43.4)	5	0.3	17.3 (7.2–41.5)	3	0.3	9.8 (3.2–30.5)
***Zambia***												
Total	131	5.2	25.1 (21.1–29.8)	87	4.9	17.6 (14.3–21.7)	72	5.1	14.0 (11.1–17.7)	63	5.2	12.0 (9.4–15.4)
*End follow-up V1-V2*	77	3.1	25.1 (20.1–31.4)	50	2.8	17.9 (13.6–23.7)	38	2.9	13.3 (9.7–18.2)	33	2.9	11.4 (8.1–16.1)
*End follow-up V2-V3*	54	2.1	25.0 (19.2–32.7)	37	2.1	17.2 (12.4–23.7)	34	2.3	15.0 (10.7–21.0)	30	2.3	12.8 (8.9–18.3)
HIV status contact*												
HIV negative	93	3.0	30.7 (25.0–37.6)	61	2.8	21.3 (16.6–27.4)	50	2.9	16.7 (12.7–22.1)	44	3.1	14.4 (10.7–19.3)
HIV positive, no ARV	31	1.9	16.1 (11.3–22.9)	20	1.9	10.7 (6.9–16.6)	16	1.9	8.4 (5.1–13.7)	15	1.9	7.8 (4.7–13.0)
HIV positive & ARV	5	0.2	20.7 (8.6–49.7)	5	0.2	24.6 (10.2–59.1)	5	0.2	23.2 (9.6–55.7)	3	0.2	12.9 (4.2–40.2)
**Region by TST prevalence**												
Lusaka, high TST	49	1.5	33.0 (24.9–43.7)	31	1.4	21.9 (15.4–31.2)	27	1.5	18.1 (12.4–26.4)	24	1.5	15.7 (10.5–23.5)
Urban, high TST	39	1.5	26.3 (19.2–35.9)	27	1.4	19.5 (13.3–28.4)	20	1.5	13.8 (8.9–21.4)	18	1.5	12.2 (7.7–19.3)
Urban, low TST	29	1.7	17.5 (12.2–25.2)	20	1.6	12.8 (8.2–19.9)	16	1.6	9.9 (6.1–16.3)	13	1.6	7.9 (4.6–13.7)
Rural, low TST	14	0.6	23.4 (13.9–39.5)	9	0.6	15.3 (7.9–29.5)	9	0.6	15.3 (7.9–29.5)	8	0.6	13.4 (6.7–26.9)
***South Africa***												
Total	44	2.3	18.9 (14.1–25.5)	27	1.9	14.3 (9.8–20.8)	21	1.9	10.8 (7.0–16.5)	16	2.0	7.9 (4.9–13.0)
*End follow-up V1-V2*	36	1.8	20.1 (14.5–27.8)	22	1.4	15.3 (10.1–23.3)	18	1.5	12.3 (7.7–19.5)	13	1.5	8.6 (4.9–14.8)
*End follow-up V2-V3*	8	0.5	15.2 (7.6–30.5)	5	0.5	10.9 (4.6–26.4)	3	0.5	6.3 (2.0–19.5)	3	0.5	6.0 (1.9–18.6)
HIV status contact*												
HIV negative	32	1.5	20.8 (14.7–29.4)	17	1.2	13.6 (8.5–21.9)	13	1.3	10.0 (5.8–17.3)	9	1.4	6.7 (3.5–12.8)
HIV positive, no ARV	11	0.7	15.2 (8.4–27.4)	10	0.6	17.6 (9.5–32.8)	8	0.6	13.6 (6.9–27.6)	7	0.6	11.9 (5.7–25.1)
HIV positive & ARV	1	0.1	17.9 (2.5–127.0)	0	0.1	N.A.	0	0.1	N.A.	0	0.1	N.A.
**TST prevalence**												
High	27	1.1	25.1 (17.2–36.7)	15	0.9	16.9 (10.2–28.1)	11	0.9	11.8 (6.6–21.4)	9	0.9	9.3 (4.8–17.8)
Low	17	1.2	13.6 (8.5–21.9)	12	1.0	11.9 (6.8–21.0)	10	1.0	9.8 (5.3–18.3)	7	1.0	6.7 (3.2–14.0)

Footnote: Contacts with visit 2 unknown and conversion at visit 3 were randomly assigned to have end point follow-up between visit 1-visit 2 (V1-V2) or visit 2-visit 3 (V2-V3). Table A4 (additional file) shows that this includes *n* = 27 household contacts if conversion definition 2 is used. Number of contacts who were randomly allocated for the other definitions: *n* = 41 definition 1; *n* = 21 definition 3; *n* = 19 definition 4 *HIV status unknown not shown

IRs and 95% CIs reported as number of conversion events per 100 person-years of follow-up time

The analysis including both countries using definition 1, showed that females were less likely than males to convert during follow-up (aRR = 0.7, 95%CI = 0.5–0.9), as were HIV positive contacts not on ARV compared to HIV negative contacts (aRR = 0.7, 95%CI = 0.4–0.9) (Table 5). A similar pattern for sex and HIV status was observed among stricter definitions but evidence of association was weaker. Contacts living in an urban, low TST prevalence area in Zambia were less likely to convert than contacts living in Lusaka, a high TST area (aRR = 0.4, 95%CI = 0.3–0.7, definition 1). This pattern remained statistically significant for stricter conversion definitions. A similar trend was visible in South Africa, but numbers are small (Table 5). Analysis using all available contact-, index-, and household characteristics did not reveal any additional associations between characteristics and QFT conversion (results not shown).

**Table 5:** Factors associated with QFT conversion at visit 2 and visit 3 using different definitions of conversion

	Definition 1 (< 0.35, increase 0.35)		Definition 2 (< 0.2, ≥0.7)		Definition 3 (< 0.2, ≥1.05)		Definition 4 (< 0.2, ≥1.4)	
	Unadjusted RR (95%CI)	Adjusted RR (95%CI)^**a**^	Unadjusted RR (95%CI)	Adjusted RR (95%CI)^**a**^	Unadjusted HR (95%CI)	Adjusted RR (95%CI)^**a**^	Unadjusted RR (95%CI)	Adjusted RR (95%CI)^**a**^
***Both countries***								
*End follow-up V1-V2*	1	1	1	1	1	1	1	1
*End follow-up V2-V3*	0.9 (0.7–1.3)	1.0 (0.8–1.4)	0.9 (0.7–1.4)	0.9 (0.7–1.4)	1.0 (0.7–1.6)	1.0 (0.7–1.6)	1.1 (0.7–1.7)	1.1 (0.7–1.8)
**Sex**								
Male	1	1	1	1	1	1	1	1
Female	0.7 (0.5–0.9)	0.7 (0.5–0.9)	0.7 (0.5–0.9)	0.7 (0.5–1.0)	0.8 (0.5–1.2)	0.8 (0.5–1.2)	0.8 (0.5–1.3)	0.8 (0.5–1.3)
**Age**								
15–24	1	1	1	1	1	1	1	1
25–29	0.6 (0.4–0.9)	0.7 (0.4–1.2)	0.6 (0.3–1.1)	0.6 (0.3–1.3)	0.4 (0.2–0.9)	0.5 (0.2–1.1)	0.4 (0.2–0.9)	0.3 (0.1–0.9)
30–34	0.6 (0.4–1.1)	0.7 (0.4–1.3)	0.6 (0.3–1.1)	0.6 (0.3–1.2)	0.7 (0.3–1.4)	0.7 (0.3–1.5)	0.6 (0.3–1.3)	0.6 (0.3–1.4)
35–39	0.7 (0.3–1.3)	0.9 (0.5–1.6)	0.6 (0.3–1.2)	0.7 (0.3–1.5)	0.7 (0.3–1.5)	0.8 (0.4–1.8)	0.7 (0.3–1.6)	0.8 (0.3–1.8)
40–49	0.9 (0.5–1.4)	1.0 (0.6–1.6)	0.7 (0.3–1.2)	0.7 (0.4–1.4)	0.5 (0.2–1.2)	0.6 (0.3–1.3)	0.3 (0.1–0.9)	0.3 (0.1–1.0)
50+	1.2 (0.8–1.8)	1.3 (0.8–2.0)	0.9 (0.5–1.7)	1.1 (0.6–1.9)	0.9 (0.5–1.7)	0.9 (0.5–1.9)	0.9 (0.5–1.8)	1.0 (0.5–2.1)
**HIV status** ^**b**^								
HIV negative	1	1	1	1	1	1	1	1
HIV positive, no ARV	0.6 (0.4–0.8)	0.7 (0.4–0.9)	0.7 (0.4–0.9)	0.8 (0.5–1.3)	0.7 (0.4–1.0)	0.8 (0.5–1.4)	0.7 (0.4–1.2)	0.9 (0.6–1.9)
HIV positive & ARV	0.7 (0.3–1.6)	0.7 (0.3–1.6)	0.9 (0.4–2.2)	1.0 (0.4–2.4)	1.2 (0.5–2.7)	1.2 (0.5–2.8)	0.8 (0.3–2.6)	0.8 (0.3–2.6)
**Region by TST prevalence**								
Zambia, Lusaka, high TST	1	1	1	1	1	1	1	1
Zambia, Urban, high TST	0.8 (0.5–1.2)	0.7 (0.4–1.0)	0.9 (0.5–1.5)	0.7 (0.4–1.2)	0.8 (0.4–1.3)	0.7 (0.4–1.2)	0.8 (0.4–1.4)	0.6 (0.3–1.1)
Zambia, Urban, low TST	0.5 (0.3–0.8)	0.5 (0.3–0.7)	0.6 (0.3–1.0)	0.5 (0.3–0.9)	0.5 (0.3–1.0)	0.5 (0.3–1.0)	0.5 (0.3–0.9)	0.4 (0.2–0.8)
Zambia, Rural, low TST	0.7 (0.4–1.3)	0.5 (0.3–1.0)	0.7 (0.3–1.5)	0.5 (0.2–1.2)	0.8 (0.4–1.9)	0.7 (0.3–1.6)	0.9 (0.4–1.9)	0.6 (0.3–1.5)
South Africa, high TST	0.8 (0.5–1.2)	0.7 (0.5–1.1)	0.8 (0.4–1.4)	0.7 (0.4–1.3)	0.7 (0.3–1.3)	0.6 (0.3–1.3)	0.6 (0.3–1.2)	0.5 (0.2–1.1)
South Africa, low TST	0.4 (0.2–0.7)	0.4 (0.2–0.6)	0.5 (0.3–1.1)	0.5 (0.3–1.0)	0.5 (0.3–1.1)	0.5 (0.2–1.2)	0.4 (0.2–0.9)	0.4 (0.2–0.9)
***Zambia***								
*End follow-up V1-V2*	1	1	1	1	1	1	1	1
*End follow-up V2-V3*	0.9 (0.7–1.4)	1.1 (0.8–1.5)	0.9 (0.6–1.4)	1.0 (0.7–1.6)	1.1 (0.7–1.8)	1.2 (0.8–1.9)	1.1 (0.7–1.8)	1.2 (0.7–1.9)
**Sex**								
Male	1	1	1	1	1	1	1	1
Female	0.7 (0.5–0.9)	0.7 (0.5–1.0)	0.7 (0.5–1.1)	0.7 (0.5–1.1)	0.8 (0.5–1.3)	0.8 (0.5–1.4)	0.9 (0.5–1.5)	0.9 (0.5–1.6)
**Age**								
15–24	1	1	1	1	1	1	1	1
25–29	0.6 (0.3–1.0)	0.7 (0.4–1.3)	0.6 (0.3–1.2)	0.7 (0.3–1.6)	0.4 (0.2–1.1)	0.5 (0.2–1.4)	0.4 (0.1–1.0)	0.4 (0.1–1.3)
30–34	0.6 (0.3–1.2)	0.7 (0.4–1.4)	0.6 (0.3–1.3)	0.7 (0.3–1.5)	0.7 (0.3–1.6)	0.8 (0.4–1.8)	0.6 (0.2–1.4)	0.6 (0.3–1.5)
35–39	0.6 (0.3–1.4)	0.8 (0.4–1.8)	0.6 (0.2–1.6)	0.8 (0.3–1.9)	0.7 (0.3–1.8)	0.9 (0.3–2.3)	0.6 (0.2–1.7)	0.7 (0.3–2.2)
40–49	0.8 (0.5–1.4)	1.0 (0.5–1.7)	0.7 (0.3–1.4)	0.8 (0.4–1.8)	0.4 (0.2–1.2)	0.5 (0.2–1.4)	0.4 (0.1–1.1)	0.4 (0.1–1.3)
50+	1.3 (0.8–2.2)	1.5 (0.9–2.5)	1.2 (0.6–2.3)	1.4 (0.7–2.7)	1.1 (0.6–2.3)	1.2 (0.6–2.6)	1.2 (0.6–2.3)	1.3 (0.6–2.7)
**HIV status**								
HIV negative	1	1	1	1	1	1	1	1
HIV positive, no ARV	0.5 (0.4–0.8)	0.6 (0.4–1.0)	0.5 (0.3–0.8)	0.6 (0.3–1.1)	0.5 (0.3–0.9)	0.6 (0.3–1.2)	0.5 (0.3–0.9)	0.7 (0.4–1.4)
HIV positive & ARV	0.7 (0.3–1.5)	0.7 (0.3–1.7)	1.2 (0.5–2.7)	1.1 (0.5–2.8)	1.4 (0.6–3.1)	1.4 (0.6–3.4)	0.9 (0.3–2.8)	0.9 (0.3–3.0)
**Region by TST prevalence**								
Lusaka, high TST	1	1	1	1	1	1	1	1
VUrban, high TST	0.8 (0.5–1.2)	0.6 (0.4–0.9)	0.9 (0.5–1.5)	0.6 (0.4–1.1)	0.8 (0.4–1.3)	0.6 (0.3–1.1)	0.8 (0.4–1.4)	0.6 (0.3–1.2)
Urban, low TST	0.5 (0.3–0.8)	0.4 (0.3–0.7)	0.6 (0.3–1.0)	0.5 (0.3–0.9)	0.5 (0.3–1.0)	0.5 (0.2–0.9)	0.5 (0.3–0.9)	0.4 (0.2–0.9)
Rural, low TST	0.7 (0.4–1.3)	0.5 (0.2–0.9)	0.7 (0.3–1.5)	0.5 (0.2–1.1)	0.8 (0.4–1.9)	0.6 (0.3–1.4)	0.9 (0.4–1.9)	0.6 (0.2–1.4)
***South Africa***								
*End follow-up V1-V2*	1	1	1	1	1	1	1	1
*End follow-up V2-V3*	0.8 (0.4–1.6)	0.8 (0.4–1.7)	0.7 (0.3–1.9)	0.8 (0.3–2.1)	0.5 (0.2–1.7)	0.6 (0.2–2.0)	0.7 (0.2–2.5)	0.9 (0.2–3.4)
**Sex**								
Male	1	1	1	1	1	1	1	1
Female	0.8 (0.4–1.6)	0.7 (0.4–1.5)	0.6 (0.3–1.5)	0.6 (0.2–1.4)	0.7 (0.3–1.7)	0.6 (0.2–1.7)	0.6 (0.2–1.7)	0.4 (0.1–1.3)
**Age**								
15–24	1	1	1	1	1	1	1	1
25–29	0.7 (0.3–1.8)	0.7 (0.3–2.2)	0.7 (0.2–1.9)	0.4 (0.1–1.7)	0.4 (0.1–1.9)	0.3 (0.1–1.6)	0.3 (0.03–2.3)	0.1 (0.01–1.7)
30–34	0.7 (0.2–2.6)	0.6 (0.1–2.7)	0.4 (0.1–2.2)	0.3 (0.1–1.8)	0.6 (0.1–2.9)	0.4 (0.1–2.5)	0.7 (0.1–3.9)	0.4 (0.1–2.7)
35–39	0.9 (0.3–2.5)	1.0 (0.4–2.9)	0.5 (0.1–2.4)	0.5 (0.1–2.1)	0.7 (0.1–3.2)	0.6 (0.1–2.8)	0.9 (0.2–4.3)	0.6 (0.2–2.7)
40–49	1.0 (0.4–2.4)	1.1 (0.4–2.8)	0.6 (0.2–2.1)	0.5 (0.1–2.1)	0.8 (0.2–2.9)	0.6 (0.1–2.9)	0.3 (0.04–2.6)	0.2 (0.01–2.2)
50+	0.9 (0.3–2.4)	0.9 (0.4–2.5)	0.4 (0.1–1.8)	0.3 (0.1–1.7)	0.3 (0.03–2.1)	0.2 (0.02–1.9)	0.3 (0.04–2.7)	0.2 (0.2–1.9)
**HIV status** ^**b**^								
HIV negative	1	1	1	1	1	1	1	1
HIV positive, no ARV	0.7 (0.4–1.5)	0.8 (0.4–1.8)	1.3 (0.6–2.9)	1.7 (0.7–4.6)	1.4 (0.6–3.4)	1.9 (0.7–5.5)	1.8 (0.6–5.0)	3.3 (0.9–12.2)
HIV positive & ARV	0.9 (0.1–6.5)	1.1 (0.1–8.6)	N.A.	N.A.	N.A.	N.A.	N.A.	N.A.
**TST prevalence**								
High	1	1	1	1	1	1	1	1
Low	0.5 (0.3–0.9)	0.5 (0.3–0.9)	0.7 (0.3–1.5)	0.8 (0.3–1.6)	0.8 (0.4–1.9)	0.9 (0.4–2.2)	0.7 (0.3–1.9)	0.9 (0.3–2.6)

^a^No rules were applied: time band, sex, age, HIV status, HH intervention (yes/no), and region by TST prevalence were simultaneously added to the regression models

^b^HIV status unknown not shown

Footnote: Contacts with visit 2 unknown and conversion at visit 3 were randomly assigned to have end point follow-up between visit 1-visit 2 or visit 2-visit 3. Table A4 (additional file) shows that this includes n = 27 household contacts if conversion definition 2 is used. Number of contacts who were randomly allocated for the other definitions: n = 41 definition 1; n = 21 definition 3; n = 19 definition 4 **HIV status unknown not shown

## Discussion

IGRA converters have a higher risk of subsequently developing active TB than those who remain negative on repeated testing [6, 17]. However, previous studies have suggested that the conversion definition should be stricter than the current manufacturers’ definition, to enable more accurate identification of recent TB infection [17–20]. In this paper we assessed the distribution of IFN-g values, using serial QFT results collected among household TB contacts, to guide identification of stricter conversion definitions, which were then used to assist the interpretation of QFT conversion results and to estimate the incidence of TB infection. We found that QFT conversion rates were relatively high, even using a more stringent definition than the manufacturer’s definition, indicating high incidence of TB infection among household TB contacts in these communities.

Our study identified three stricter conversion definitions to estimate the incidence of TB infection. Each conversion definition included a mandatory absolute increase between baseline and follow-up QFT measurements, varying from 0.5 to 1.2 IU/ml. We found that sex of contact, HIV status and TST prevalence region, all well established risk factors of TB infection risk, were associated with QFT conversion. With stricter conversion definitions, associations between aforementioned risk factors and conversion continued to show similar patterns. Our strictest definition plausibly has high specificity. However, the trade-off between sensitivity and specificity using different thresholds of QFT conversion remains unknown, due to the absence of a reference standard, and because we could not identify clear “breakpoints” in the distribution of IFN-g response.

Irrespective of which conversion definition was used, incidence of TB infection estimates were high, at 14.4–30.7 per 100 person years in Zambia and 6.7–20.8 in South Africa, measured among HIV negative TB contacts. These rates were much higher than found in previous studies in South Africa estimating the annual risk of tuberculosis infection (ARTI) [24, 26–28]. The ARTI is considered the best epidemiological indicator to measure the extent of TB transmission at community level [29]. Previous TST surveys conducted among children in high TB incidence communities in South Africa found an ARTI of 4% [24, 26, 27]. Dodd et al. modelled TB infection incidence among adults based on data from a social contact pattern survey and TST prevalence survey, conducted in the same South African and Zambian communities as our study [28]. The ARTI was estimated at 6–8% for females and 7–10% for males in South Africa and 2–5% for females and 3–7% for males in Zambia.

The relatively higher incidence estimates found in our study suggest that, despite high background rates of infection in the general community, the risk of acquiring infection with *M. tuberculosis* is still higher among household members of TB patients. Consistent with our findings, Verver et al. showed that household contacts of TB patients still have a higher risk of being infected with the same *M. tuberculosis* strain as the index patient than do community contacts in South Africa [30]. Thus, while previous evidence has suggested a substantial role for transmission outside the household [31–33], it remains important to consider household transmission when designing interventions to reduce the incidence of new infection and to reduce the risk of infection progressing to disease.

TB infection prevalence estimates in our study, measured by QFT positivity at baseline, were higher in South Africa than in Zambia (67–82% and 55–65%, respectively among HIV negative contacts). The 2005 TST prevalence survey, conducted at baseline of the ZAMSTAR trial, also reported higher TB infection prevalence in South Africa compared to Zambia [22]. Indeed, South Africa is still among the countries with the highest TB incidence in the world [34], and the communities involved in this study were high-density residential areas [22]. In contrast to the infection prevalence at baseline, the incidence rates among household contacts of TB patients in our study were higher in Zambia compared with South Africa. This might be the result of a local saturation of susceptible individuals. Infected individuals are generally linked to other infected individuals, by whom they were infected or to whom they have transmitted infection [35]. As a result, the number of contacts between infected and susceptible individuals is reduced over time and the spread of infection slowed. This might explain the greater proportion with TB infection at baseline compared to the smaller proportion with new infections during follow-up in South African households, and the other way around in Zambian TB households. It might also reflect different living conditions in the Zambian and South African communities, and/or differences among the TB index patients in the time from developing TB disease to being diagnosed with TB and starting TB treatment.

TB infection prevalence and incidence were considerably lower among HIV positive contacts on ARV compared to HIV negative contacts. This is probably the result of immunosuppression by HIV infection on the antigen response which can result in a low negative predictive value of the IGRA in HIV positive individuals [25]. Accurate identification of TB infection among immunocompromised patients significantly diminishes the risk of developing active TB if they are put on preventive treatment, so more accurate diagnostic tests for TB infection would be valuable. The recently FDA-cleared QuantiFERON-TB Gold Plus has been proposed to improve the detection of TB infection in immunocompromised patients through stimulation of CD8^+^ T cells [36–38]. However, initial studies show high overall agreement with the QFT-GIT, suggesting a minimal difference in assay performance [37, 39, 40].

This study had several limitations. First, incidence of TB infection estimates in this study of household contacts were based on QFT outcomes, and they were compared to ARTI estimates for the general population that were made from community TST surveys. We cannot exclude the possibility that differences in estimates found in our study are partly the result of different tests used, and it has previously been shown that there was considerable discordance between the QFT and TST response at baseline in the same communities as our study [41]. Second, we might have overestimated the amount of transmission attributable to household transmission. We found that conversion rates were fairly similar between visit 2 and 3 compared to visit 1 and 2. Whether this persistent risk reflects household transmission, ongoing community transmission, clustering of risk factors within TB-affected households, reactivation of latent TB infection, or a combination of factors, our findings emphasise the need for increased case detection in these study communities. Further research is required to determine whether active-, passive- or community-wide TB case finding is most effective in reducing the prevalence of TB in these communities. Future studies assessing QFT conversion should aim to follow individuals up for at least several years, to allow TB progression after conversion. Also, if *M. tuberculosis* genotyping was done for both the index patient and a household contact who subsequently developed TB, this would help to distinguish within-household versus community transmission and enable more accurate estimates of household transmission. Finally, since we only started testing with QFT half way through recruitment of household contacts we had to exclude a substantial proportion of eligible contacts from our study. However, we do not expect that this resulted in bias since the timing of when households were enrolled depended only on when an index TB patient was diagnosed at the clinic.

## Conclusions

This study found high QFT conversion rates even with the strictest conversion definition, indicating high incidence of TB infection among household contacts of TB patients in South African and Zambian communities. The boundaries of more to less strict definitions of QFT conversion provided in this study can assist the interpretation of serial QFT outcomes and to estimate TB infection incidence in comparable settings. These findings add to the limited evidence on the performance of serial IGRA testing in large longitudinal studies. Future studies should evaluate the proposed stringent conversion definitions in different settings and populations to determine its application to target individuals for preventive TB treatment. Until then, serial IGRA results should be interpreted with caution. Individuals who convert according to the manufacturers’ definition should be closely monitored to confirm conversion at a later time, especially low-risk individuals.

## Supplementary information

**Additional file 1 Fig. A1.** Distribution IFN-gamma results among 57 household contacts who developed tuberculosis during follow-up. * The most recently available QFT test result prior to TB diagnosis was used. For *n* = 40 contacts who developed TB between V1-V2, this was QFT test result at visit 1. For *n* = 17 contacts who developed TB between V2-V3, this was QFT test result at visit 2 (*n* = 12), or at visit 1 when visit 2 QFT test result was missing (*n* = 5). ** Histogram was plotted among both HIV negative and HIV positive household contacts.

**Additional file 2 Fig. A2**. Distribution IFN-gamma results among 1165 household contacts who did not develop tuberculosis during follow-up. * QFT test result at visit 1 was used for the 1165 contacts who did not develop TB during follow-up. ** Histogram was plotted among both HIV negative and HIV positive household contacts

**Additional file 3 Table A1.** Factors associated with a positive QFT result at visit 1 using definition 2 (≥0.35 IU/ml). * sex, age, HIV status, HH intervention (yes/no), and region by TST prevalence were simultaneously added to the regression models ** Unknown HIV status not shown

**Additional file 4 Table A2**. Characteristics of household contacts by incident tuberculosis status. ^1^ Pearson’s chi-squared test, unless stated otherwise. ^2^ Two-sample Wilcoxon rank-sum (Mann-Whitney) test. * The most recently available QFT test result prior to TB diagnosis was used. For n = 40 contacts who developed TB between V1-V2, this was QFT test result at visit 1. For n = 17 contacts who developed TB between V2-V3, this was QFT test result at visit 2 (n = 12), or at visit 1 when visit 2 QFT test result was missing (n = 5). ** QFT test result at visit 1 was used for the 1165 contacts who did not develop TB during follow-up.

**Additional file 5 Table A3**. Index patient characteristics associated with a positive QFT result at visit 1 using definition 2 (≥0.35 IU/ml). *Regression models were constructed using forward selection as described in Methods, using all available contact-, index-, and household characteristics. Only index factors associated with outcome were presented.

**Additional file 6 Table A4** Study population QFT conversion analysis using conversion definition 2 (< 0.2, ≥0.7). ^**a**^ End point follow-up was placed halfway visits for contacts who converted, and was the date of the last negative QFT measurement for contacts who did not convert. To account for uncertainty between the follow-up QFT measurements, analysis time was split into visit 1-visit 2 and visit 2-visit 3. ^**b**^ A random variable allocated approximately 50% of contacts with unknown visit 2 status and conversion at visit 3, to have end point follow-up halfway visit 1-visit 2 and ~ 50% half-way visit 2-visit 3. This was informed by the distribution of QFT conversion between visit 1–2 and visit 2–3 among contacts with an available QFT measurement at visit 1, 2, and 3.

**Additional file 7 Table A5**. Incidence rate QFT conversion at visit 2 and visit 3 using different definitions of conversion. *This analysis is restricted to household contacts who have a known visit 2 QFT result.*

## Data Availability

The datasets used and/or analysed during the current study are available from the corresponding author on reasonable request.

## Abbreviations

TB: Tuberculosis
LTBI: Latent tuberculosis infection
TST: Tuberculin skin test
IFN-g: Interferon gamma
IGRA: Interferon gamma release assays
QFT: QuantiFERON-TB Gold In-Tube
CDC: Centers for Disease Control and Prevention
ZAMSTAR: Zambia South Africa tuberculosis and AIDS Reduction
ECF: Enhanced case finding
HH: Household
TBAg: TB antigen
Nil: Negative control response
OR: Odds ratio
IR: Incidence rate
CI: Confidence interval
RR: Rate ratio
ARTI: Annual risk of tuberculosis infection
