# Supplementary figures and images for: Interpretation of serial interferon-gamma test results to measure new tuberculosis infection among household contacts in Zambia and South Africa
Source: BMC Infect Dis. 2020 Oct 15;20:760. doi: 10.1186/s12879-020-05483-9 (PMC7559914; doi:10.1186/s12879-020-05483-9)

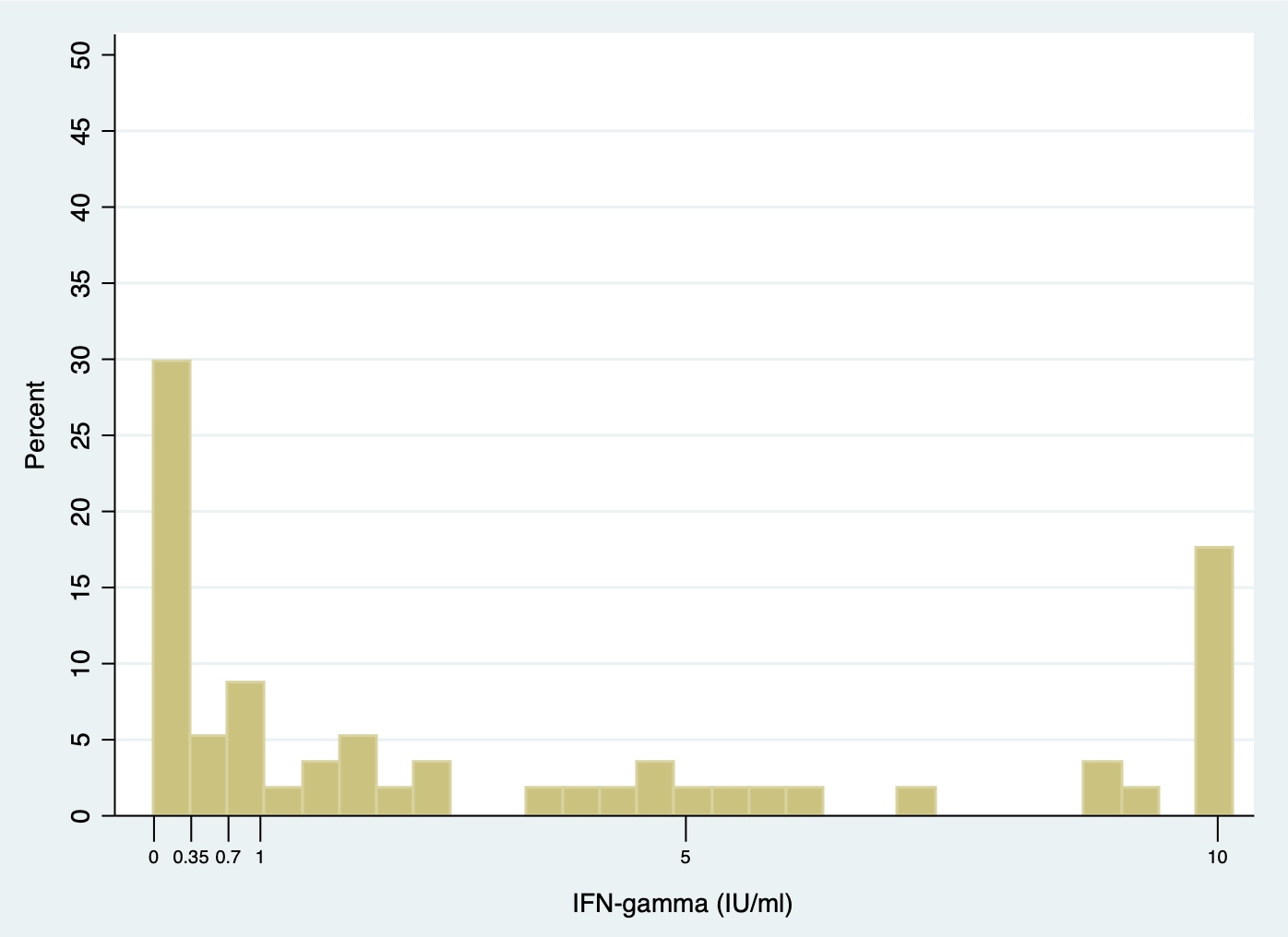

Supplement: Supplementary file 1 — Additional file 1 Fig. A1. Distribution IFN-gamma results among 57 household contacts who developed tuberculosis during follow-up. * The most recently available QFT test result prior to TB diagnosis was used. For n = 40 contacts who developed TB between V1-V2, this was QFT test result at visit 1. For n = 17 contacts who developed TB between V2-V3, this was QFT test result at visit 2 (n = 12), or at visit 1 when visit 2 QFT test result was missing (n = 5). ** Histogram was plotted among both HIV negative and HIV positive household contacts. [file 12879_2020_5483_MOESM1_ESM.jpg]

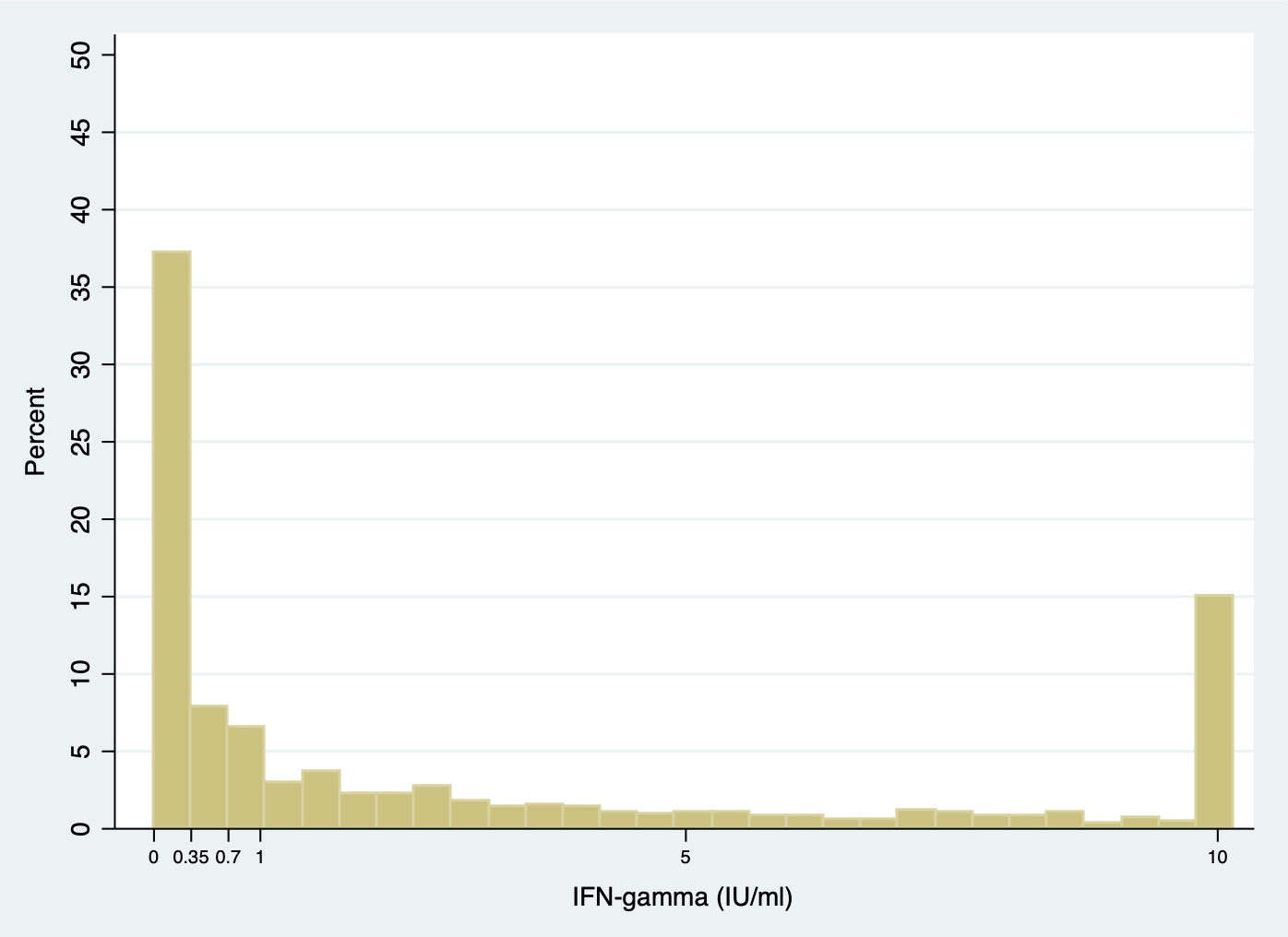

Supplement: Supplementary file 2 — Additional file 2 Fig. A2. Distribution IFN-gamma results among 1165 household contacts who did not develop tuberculosis during follow-up. * QFT test result at visit 1 was used for the 1165 contacts who did not develop TB during follow-up. ** Histogram was plotted among both HIV negative and HIV positive household contacts [file 12879_2020_5483_MOESM2_ESM.jpg]
