# Supplementary material for: Interpretation of serial interferon-gamma test results to measure new tuberculosis infection among household contacts in Zambia and South Africa
Source: BMC Infect Dis. 2020 Oct 15;20:760. doi: 10.1186/s12879-020-05483-9 (PMC7559914; doi:10.1186/s12879-020-05483-9)
Supplement: Supplementary file 3 — Additional file 3 Table A1. Factors associated with a positive QFT result at visit 1 using definition 2 (≥0.35 IU/ml). * sex, age, HIV status, HH intervention (yes/no), and region by TST prevalence were simultaneously added to the regression models ** Unknown HIV status not shown [file 12879_2020_5483_MOESM3_ESM.docx]

**Table A1. Factors associated with a positive QFT result at visit 1 using definition 2 (≥0.35 IU/ml)**

|  | **QFT positive *n* (%)** | **total (n)** | **Unadjusted OR (95%CI)** | **p-value** | **Adjusted**  **OR (95%CI)*** | **p-value** |
| --- | --- | --- | --- | --- | --- | --- |
| ***Both countries*** | |  |  |  |  |  |
| **Sex** |  |  |  |  |  |  |
| Male | 368 (62) | 591 | 1 |  | 1 |  |
| Female | 875 (64) | 1,377 | 1.1 (0.9-1.3) | 0.590 | 1.1 (0.9-1.3) | 0.439 |
| **Age** |  |  |  |  |  |  |
| 15-24 | 447 (59) | 754 | 1 |  | 1 |  |
| 25-29 | 181 (64) | 284 | 1.2 (0.9-1.6) | 0.185 | 1.5 (1.1-2.0) | 0.010 |
| 30-34 | 130 (60) | 215 | 1.1 (0.8-1.4) | 0.756 | 1.3 (0.9-1.9) | 0.087 |
| 35-39 | 106 (70) | 151 | 1.6 (1.1-2.4) | 0.015 | 1.9 (1.3-2.9) | 0.002 |
| 40-49 | 155 (67) | 233 | 1.4 (1.0-1.9) | 0.048 | 1.4 (1.0-1.9) | 0.030 |
| 50+ | 224 (68) | 331 | 1.4 (1.1-1.9) | 0.011 | 1.4 (1.0-1.8) | 0.034 |
| **HIV status** | |  |  |  |  |  |
| HIV negative | 942 (67) | 1,402 | 1 |  | 1 |  |
| HIV positive, no ARV | 258 (53) | 491 | 0.5 (0.4-0.7) | <0.001 | 0.5 (0.4-0.6) | <0.001 |
| HIV positive & ARV | 28 (51) | 55 | 0.5 (0.3-0.9) | 0.020 | 0.4 (0.3-0.8) | 0.006 |
| Unknown | 15 (75) | 20 |  |  |  |  |
| **Region by TST prevalence** | | |  |  |  |  |
| Zambia, Lusaka, high TST | 234 (64) | 363 | 1 |  | 1 |  |
| Zambia, Urban, high TST | 186 (57) | 326 | 0.7 (0.5-1.0) | 0.079 | 0.7 (0.5-0.9) | 0.045 |
| Zambia, Urban, low TST | 181 (52) | 350 | 0.6 (0.4-0.8) | 0.001 | 0.5 (0.4-0.8) | <0.001 |
| Zambia, Rural, low TST | 68 (54) | 125 | 0.7 (0.4-1.0) | 0.075 | 0.6 (0.4-0.9) | 0.022 |
| South Africa, high TST | 338 (74) | 457 | 1.6 (1.2-2.1) | 0.004 | 1.4 (1.0-1.9) | 0.032 |
| South Africa, low TST | 236 (68) | 347 | 1.2 (0.8-1.6) | 0.335 | 1.1 (0.8-1.5) | 0.738 |
| ***Zambia*** |  |  |  |  |  |  |
| **Sex** |  |  |  |  |  |  |
| Male | 203 (54) | 374 | 1 |  | 1 |  |
| Female | 466 (59) | 790 | 1.2 (0.9-1.6) | 0.128 | 1.2 (0.9-1.6) | 0.096 |
| **Age** |  |  |  |  |  |  |
| 15-24 | 239 (52) | 459 | 1 |  | 1 |  |
| 25-29 | 107 (61) | 175 | 1.4 (1.0-2.1) | 0.039 | 1.7 (1.2-2.6) | 0.005 |
| 30-34 | 76 (56) | 135 | 1.2 (0.8-1.7) | 0.388 | 1.5 (0.9-2.2) | 0.074 |
| 35-39 | 55 (64) | 86 | 1.6 (1.0-2.7) | 0.049 | 2.0 (1.2-3.4) | 0.006 |
| 40-49 | 72 (61) | 118 | 1.4 (0.9-2.2) | 0.084 | 1.7 (1.1-2.6) | 0.023 |
| 50+ | 120 (63) | 191 | 1.6 (1.1-2.2) | 0.015 | 1.6 (1.1-2.3) | 0.007 |
| **HIV status** | |  |  |  |  |  |
| HIV negative | 484 (61) | 797 | 1 |  | 1 |  |
| HIV positive, no ARV | 158 (50) | 316 | 0.6 (0.5-0.8) | 0.001 | 0.5 (0.4-0.7) | <0.001 |
| HIV positive & ARV | 16 (43) | 37 | 0.5 (0.2-0.9) | 0.047 | 0.4 (0.2-0.8) | 0.008 |
| Unknown | 11 (79) | 14 |  |  |  |  |
| **TST prevalence region** | | |  |  |  |  |
| Lusaka, high TST | 234 (64) | 363 | 1 |  | 1 |  |
| Urban, high TST | 186 (57) | 326 | 0.7 (0.5-1.0) | 0.079 | 0.6 (0.4-0.9) | 0.014 |
| Urban, low TST | 181 (52) | 350 | 0.6 (0.4-0.8) | 0.001 | 0.5 (0.4-0.7) | <0.001 |
| Rural, low TST | 68 (54) | 125 | 0.7 (0.4-1.0) | 0.075 | 0.6 (0.3-0.9) | 0.016 |
| ***South Africa*** | |  |  |  |  |  |
| **Sex** |  |  |  |  |  |  |
| Male | 165 (76) | 217 | 1 |  | 1 |  |
| Female | 409 (70) | 587 | 0.7 (0.5-1.1) | 0.094 | 0.8 (0.6-1.2) | 0.294 |
| **Age** |  |  |  |  |  |  |
| 15-24 | 208 (71) | 295 | 1 |  | 1 |  |
| 25-29 | 74 (68) | 109 | 0.9 (0.6-1.4) | 0.600 | 1.1 (0.7-1.8) | 0.729 |
| 30-34 | 54 (68) | 80 | 0.9 (0.5-1.5) | 0.611 | 1.1 (0.6-1.9) | 0.753 |
| 35-39 | 51 (78) | 65 | 1.5 (0.8-2.9) | 0.206 | 1.8 (0.9-3.6) | 0.091 |
| 40-49 | 83 (72) | 115 | 1.1 (0.7-1.7) | 0.734 | 1.1 (0.7-1.8) | 0.659 |
| 50+ | 104 (74) | 140 | 1.2 (0.8-1.9) | 0.417 | 1.1 (0.7-1.7) | 0.794 |
| **HIV status** | |  |  |  |  |  |
| HIV negative | 458 (76) | 605 | 1 |  | 1 |  |
| HIV positive, no ARV | 100 (57) | 175 | 0.4 (0.3-0.6) | <0.001 | 0.4 (0.3-0.6) | <0.001 |
| HIV positive & ARV | 12 (67) | 18 | 0.6 (0.2-1.8) | 0.395 | 0.7 (0.2-2.0) | 0.500 |
| Unknown | 4 (67) | 6 |  |  |  |  |
| **TST prevalence region** | | |  |  |  |  |
| High | 338 (74) | 457 | 1 |  | 1 |  |
| Low | 236 (68) | 347 | 0.7 (0.5-1.0) | 0.075 | 0.7 (0.5-1.0) | 0.076 |

* sex, age, HIV status, HH intervention (yes/no), and region by TST prevalence were simultaneously added to the regression models ** Unknown HIV status not shown
