# Supplementary material for: Interpretation of serial interferon-gamma test results to measure new tuberculosis infection among household contacts in Zambia and South Africa
Source: BMC Infect Dis. 2020 Oct 15;20:760. doi: 10.1186/s12879-020-05483-9 (PMC7559914; doi:10.1186/s12879-020-05483-9)
Supplement: Supplementary file 4 — Additional file 4 Table A2. Characteristics of household contacts by incident tuberculosis status. 1 Pearson’s chi-squared test, unless stated otherwise. 2 Two-sample Wilcoxon rank-sum (Mann-Whitney) test. * The most recently available QFT test result prior to TB diagnosis was used. For n = 40 contacts who developed TB between V1-V2, this was QFT test result at visit 1. For n = 17 contacts who developed TB between V2-V3, this was QFT test result at visit 2 (n = 12), or at visit 1 when visit 2 QFT test result was missing (n = 5). ** QFT test result at visit 1 was used for the 1165 contacts who did not develop TB during follow-up. [file 12879_2020_5483_MOESM4_ESM.docx]

**Table A2. Characteristics of household contacts by incident tuberculosis status**

|  | **Incident TB**  ***n* (%)** | **No incident TB**  ***n* (%)** | **p-value^1^** |
| --- | --- | --- | --- |
| *Total* | 57 | 1,165 |  |
| **Sex** |  |  |  |
| Male | 16 (28) | 331 (28) | 0.955 |
| Female | 41 (72) | 834 (72) |  |
| **Age** |  |  |  |
| 15-24 | 12 (21) | 440 (38) | 0.001 |
| 25-29 | 15 (26) | 146 (13) |  |
| 30-34 | 12 (21) | 114 (10) |  |
| 35-39 | 4 (7) | 96 (8) |  |
| 40-49 | 7 (12) | 151 (13) |  |
| 50+ | 7 (12) | 218 (19) |  |
| **HIV status** |  |  |  |
| HIV negative | 19 (33) | 862 (74) | <0.001 |
| HIV positive, no ARV | 32 (56) | 261 (22) |  |
| HIV positive & ARV | 6 (11) | 29 (2) |  |
| Unknown | 0 | 13 (1) |  |
| **QFT outcome (IFN-g)** |  |  |  |
| Median (IQR) | 1.8 (0.2-6.2)* | 1.0 (0.1-5.4)** | 0.109^2^ |

^1^ Pearson's chi-squared test, unless stated otherwise

^2^ Two-sample Wilcoxon rank-sum (Mann-Whitney) test.

* The most recently available QFT test result prior to TB diagnosis was used. For n=40 contacts who developed TB between V1-V2, this was QFT test result at visit 1. For n=17 contacts who developed TB between V2-V3, this was QFT test result at visit 2 (n=12), or at visit 1 when visit 2 QFT test result was missing (n=5).

** QFT test result at visit 1 was used for the 1,165 contacts who did not develop TB during follow-up.
