# Supplementary material for: Interpretation of serial interferon-gamma test results to measure new tuberculosis infection among household contacts in Zambia and South Africa
Source: BMC Infect Dis. 2020 Oct 15;20:760. doi: 10.1186/s12879-020-05483-9 (PMC7559914; doi:10.1186/s12879-020-05483-9)
Supplement: Supplementary file 5 — Additional file 5 Table A3. Index patient characteristics associated with a positive QFT result at visit 1 using definition 2 (≥0.35 IU/ml). *Regression models were constructed using forward selection as described in Methods, using all available contact-, index-, and household characteristics. Only index factors associated with outcome were presented. [file 12879_2020_5483_MOESM5_ESM.docx]

**Table A3. Index patient characteristics associated with a positive QFT result at visit 1 using definition 2 (≥0.35 IU/ml)**

|  | **Unadjusted OR (95%CI)** | **p-value** | **Adjusted OR* (95%CI)** | **p-value** |
| --- | --- | --- | --- | --- |
| **Both countries** |  |  |  |  |
| Index TB status |  |  |  |  |
| Pulmonary smear positive | 1 |  | 1 |  |
| Pulmonary smear negative | 0.7 (0.6-0.9) | 0.016 | 0.9 (0.7-1.1) | 0.324 |
| Extra pulmonary | 0.6 (0.4-0.8) | 0.001 | 0.6 (0.4-0.9) | 0.005 |
| **Zambia** |  |  |  |  |
| Index TB status |  |  |  |  |
| Pulmonary smear positive | 1 |  | 1 |  |
| Pulmonary smear negative | 0.7 (0.5-0.9) | 0.032 | 0.7 (0.5-0.9) | 0.031 |
| Extra pulmonary | 0.5 (0.4-0.8) | 0.004 | 0.5 (0.3-0.8) | 0.006 |

*Regression models were constructed using forward selection as described in Methods, using all available contact-, index-, and household characteristics. Only index factors associated with outcome were presented.
