# Supplementary material for: Interpretation of serial interferon-gamma test results to measure new tuberculosis infection among household contacts in Zambia and South Africa
Source: BMC Infect Dis. 2020 Oct 15;20:760. doi: 10.1186/s12879-020-05483-9 (PMC7559914; doi:10.1186/s12879-020-05483-9)
Supplement: Supplementary file 6 — Additional file 6 Table A4 Study population QFT conversion analysis using conversion definition 2 (< 0.2, ≥0.7). a End point follow-up was placed halfway visits for contacts who converted, and was the date of the last negative QFT measurement for contacts who did not convert. To account for uncertainty between the follow-up QFT measurements, analysis time was split into visit 1-visit 2 and visit 2-visit 3. b A random variable allocated approximately 50% of contacts with unknown visit 2 status and conversion at visit 3, to have end point follow-up halfway visit 1-visit 2 and ~ 50% half-way visit 2-visit 3. This was informed by the distribution of QFT conversion between visit 1–2 and visit 2–3 among contacts with an available QFT measurement at visit 1, 2, and 3. [file 12879_2020_5483_MOESM6_ESM.docx]

**Table A4. Study population QFT conversion analysis using conversion definition 2 (<0.2, ≥0.7)**

| **Country** | **QFT status at visit 1** | **QFT status at visit 2** | **QFT status at visit 3** | **Conversion status** | **End point follow-up^a^** |
| --- | --- | --- | --- | --- | --- |
|  | Negative | Positive | Positive | Converter at visit 2 | Halfway visit 1- visit 2 |
| Zambia | 445 | 15 | 15 |  |  |
| South Africa | 182 | 4 | 4 |  |  |
|  | Negative | Positive | Negative | Converter at visit 2 | Halfway visit 1- visit 2 |
| Zambia | 445 | 5 | 5 |  |  |
| South Africa | 182 | 3 | 3 |  |  |
|  | Negative | Positive | Unknown | Converter at visit 2 | Halfway visit 1- visit 2 |
| Zambia | 445 | 21 | 21 |  |  |
| South Africa | 182 | 13 | 13 |  |  |
|  | Negative | Negative | Positive | Converter at visit 3 | Halfway visit 2- visit 3 |
| Zambia | 445 | 24 | 24 |  |  |
| South Africa | 182 | 2 | 2 |  |  |
|  | Negative | Negative | Negative | Non-converter at visit 3 | Exit at visit 3 |
| Zambia | 445 | 88 | 88 |  |  |
| South Africa | 182 | 22 | 22 |  |  |
|  | Negative | Negative | Unknown | Non-converter at visit 2 | Exit at visit 2 |
| Zambia | 445 | 80 | 80 |  |  |
| South Africa | 182 | 22 | 22 |  |  |
|  | Negative | Unknown | Positive | Converter at visit 3 | Random allocation halfway visit 1- visit 2, visit 2- visit 3^b^ |
| Zambia | 445 | 22 | 22 |  |  |
| South Africa | 182 | 5 | 5 |  |  |
|  | Negative | Unknown | Negative | Non-converter at visit 3 | Exit at visit 3 |
| Zambia | 445 | 27 | 27 |  |  |
| South Africa | 182 | 21 | 21 |  |  |
|  | Negative | Unknown | Unknown | Excluded from analysis |  |
| Zambia | 445 | 163 | 163 |  |  |
| South Africa | 182 | 90 | 90 |  |  |

**^a^** End point follow-up was placed halfway visits for contacts who converted, and was the date of the last negative QFT measurement for contacts who did not convert. To account for uncertainty between the follow-up QFT measurements, analysis time was split into visit 1-visit 2 and visit 2-visit 3.

**^b^** A random variable allocated approximately 50% of contacts with unknown visit 2 status and conversion at visit 3, to have end point follow-up halfway visit 1-visit 2 and ~50% half-way visit 2-visit 3. This was informed by the distribution of QFT conversion between visit 1-2 and visit 2-3 among contacts with an available QFT measurement at visit 1, 2, and 3.
