# Supplementary material for: Interpretation of serial interferon-gamma test results to measure new tuberculosis infection among household contacts in Zambia and South Africa
Source: BMC Infect Dis. 2020 Oct 15;20:760. doi: 10.1186/s12879-020-05483-9 (PMC7559914; doi:10.1186/s12879-020-05483-9)
Supplement: Supplementary file 7 — Additional file 7 Table A5. Incidence rate QFT conversion at visit 2 and visit 3 using different definitions of conversion. This analysis is restricted to household contacts who have a known visit 2 QFT result. [file 12879_2020_5483_MOESM7_ESM.docx]

**Table A5. Incidence rate QFT conversion at visit 2 and visit 3 using different definitions of conversion**

*This analysis is restricted to household contacts who have a known visit 2 QFT result*

|  | **Definition 1**  (<0.35, increase 0.35) | **Definition 2**  (<0.2, ≥0.7) | **Definition 3**  (<0.2, ≥1.05) | **Definition 4**  (<0.2, ≥1.4) |
| --- | --- | --- | --- | --- |
|  | **Incident Rate**  **(95%CI)** | **Incident Rate (95%CI)** | **Incident Rate (95%CI)** | **Incident Rate (95%CI)** |
| ***Both countries*** |  |  |  |  |
| Total | 24.4 (20.7-28.8) | 17.0 (13.8-21.0) | 13.7 (10.9-17.3) | 11.2 (8.7-14.4) |
| *End follow-up V1-V2* | 25.9 (21.4-31.5) | 18.8 (14.6-24.2) | 14.7 (11.1-19.5) | 11.5 (8.4-15.7) |
| *End follow-up V2-V3* | 21.3 (15.8-28.9) | 14.0 (9.5-20.6) | 12.0 (7.9-18.1) | 10.6 (6.9-16.2) |
| **HIV status contact** |  |  |  |  |
| HIV negative | 28.5 (23.6-34.5) | 19.6 (15.3-25.0) | 15.7 (11.9-20.6) | 12.6 (9.3-16.9) |
| HIV positive, no ARV | 16.5 (11.6-23.5) | 12.1 (7.8-18.8) | 9.6 (5.9-15.6) | 8.2 (4.9-13.9) |
| HIV positive & ARV | 20.2 (8.4-48.6) | 17.7 (6.6-47.1) | 16.7 (6.3-44.6) | 12.1 (3.9-37.7) |
| ***Zambia*** |  |  |  |  |
| Total | 24.9 (20.6-30.2) | 16.6 (13.1-21.3) | 13.5 (10.3-17.6) | 11.5 (8.7-15.4) |
| *End follow-up V1-V2* | 26.7 (21.0-33.8) | 18.0 (13.3-24.5) | 13.7 (9.7-19.4) | 11.5 (7.9-16.8) |
| *End follow-up V2-V3* | 22.3 (16.1-30.8) | 14.8 (9.9-22.0) | 13.1 (8.6-19.9) | 11.6 (7.5-17.9) |
| **HIV status contact** |  |  |  |  |
| HIV negative | 30.0 (24.0-37.5) | 20.4 (15.4-26.9) | 16.1 (11.8-21.9) | 13.9 (9.9-19.3) |
| HIV positive, no ARV | 15.9 (10.5-24.2) | 9.2 (5.2-16.1) | 7.6 (4.1-14.1) | 6.7 (3.5-12.9) |
| HIV positive & ARV | 18.6 (6.9-49.6) | 22.6 (8.5-60.3) | 21.1 (7.9-56.3) | 15.2 (4.9-47.1) |
| **Region by TST prevalence** | |  |  |  |
| Lusaka, high TST | 35.3 (25.5-48.9) | 21.4 (13.8-33.2) | 18.7 (11.8-29.6) | 16.1 (9.9-26.3) |
| Urban, high TST | 25.1 (17.7-35.5) | 18.9 (12.5-28.8) | 14.3 (8.9-22.9) | 12.3 (7.4-20.4) |
| Urban, low TST | 17.4 (11.7-25.9) | 11.7 (7.1-19.4) | 8.3 (4.6-14.9) | 6.0 (3.0-12.0) |
| Rural, low TST | 24.2 (14.1-41.7) | 15.2 (7.6-30.4) | 15.2 (7.6-30.4) | 15.2 (7.6-30.4) |
| ***South Africa*** |  |  |  |  |
| Total | 23.0 (16.8-31.6) | 18.3 (12.1-27.8) | 14.6 (9.2-23.2) | 9.9 (5.8-17.2) |
| *End follow-up V1-V2* | 24.6 (17.5-34.6) | 20.6 (13.3-31.9) | 17.0 (10.6-27.4) | 11.5 (6.5-20.2) |
| *End follow-up V2-V3* | 16.3 (6.8-39.1) | 8.7 (2.2-34.7) | 4.3 (0.6-30.3) | 3.9 (0.6-27.8) |
| **HIV status contact** |  |  |  |  |
| HIV negative | 25.0 (17.3-36.2) | 17.2 (10.2-29.1) | 14.4 (8.2-25.4) | 8.9 (4.5-17.9) |
| HIV positive, no ARV | 18.0 (9.4-34.7) | 23.5 (11.8-47.1) | 17.1 (7.7-37.9) | 13.9 (5.8-33.5) |
| HIV positive & ARV | 31.3 (4.4-222.3) | N.A. | N.A. | N.A. |
| **TST prevalence** |  |  |  |  |
| High | 31.3 (20.6-47.6) | 22.2 (12.3-40.1) | 17.5 (9.1-33.7) | 12.6 (6.0-26.5) |
| Low | 16.9 (10.3-27.5) | 15.6 (8.6-28.1) | 12.5 (6.5-24.1) | 8.0 (3.6-17.9) |
